# Octreotide in the Control of Post-Sclerotherapy Bleeding from Oesophageal Varices, Ulcers and Oesophagitis

**DOI:** 10.1155/1996/39486

**Published:** 1996

**Authors:** Spencer A. Jenkins, Andrew N. Kingsnorth, Simon Ellenbogen, Graham Copeland, Nicholas Davies, Robert Sutton, Robert Shields

**Affiliations:** 1 University Department of Surgery Royal Liverpool University Hospital Prescot Street Liverpool L7 8XP UK; 2 Academic Department of Surgery Postgraduate Medical School Morriston Hospital Swansea SA6 6NL United Kingdom

## Abstract

Bleeding from oesophageal varices, oesophageal ulcers or oesophagitis is occasionally massive and difficult to control. Octreotide, a synthetic analogue of somatostin lowers portal pressure and collateral blood flow including that through varices, increases lower oesophageal sphincter pressure, and inhibits the gastric secretion of acid as well as pepsin. Our current experience suggests it is effective in controlling acute variceal haemorrhage. Therefore we have examined the efficacy of octreotide in the control of postsclerotherapy bleeding from oesophageal varices, oesophageal ulcers and oesophagitis. During the study period 77 patients experienced a significant gastrointestinal bleed (blood pressure < 100 mm Hg, pulse > 100 beats per min or the need to transfuse 2 or more units of blood to restore the haemoglobin level) following injection sclerotherapy of oesophageal varices. The source of bleeding was varices in 42 patients, oesophageal ulcers in 31 and oesophagitis in 4. All patients received a continuous intravenous infusion of octreotide (50 μg/h) for between 40–140h. If bleeding was not controlled in the first 12h after commencing octreotide hourly bolus doses (50 μg) for 24h were superimposed on the continuous infusion. Haemorrhage was successfully controlled by an infusion of octreotide in 38 of the 42 patients with bleeding from varices, in 30 of 31 patients with oesophageal ulceration, and all patients with oesophagitis. In the 1 patient with persistent bleeding from oesophageal ulceration and in 2 of the 4 with continued haemorrhage from varices, haemostasis was achieved by hourly boluses of 50 μg octreotide for 24h in addition to the continuous infusion. No major complications were associated with octreotide administration. The results of this study clearly indicate that octreotide is a safe and effective treatment for the  control of severe haemorrhage after technically successful injection sclerotherapy.


*HPB Surgery*, 1996, Vol.10, pp. 1–6
Reprints available directly from the publisher
Photocopying permitted by license only

© 1996 OPA (Overseas Publishers Association)
Amsterdam B.V. Published in The Netherlands
by Harwood Academic Publishers GmbH
Printed in Malaysia

### Octreotide in the Control of Post-Sclerotherapy Bleeding from Oesophageal Varices, Ulcers and Oesophagitis

SPENCER A. JENKINS, ANDREW N. KINGSNORTH, SIMON ELLENBOGEN, GRAHAM COPELAND, NICHOLAS DAVIES, ROBERT SUTTON and ROBERT SHIELDS

University Department of Surgery, Royal Liverpool University Hospital, Prescot Street, Liverpool, L7 8XP, UK

*(Received 1 November 1995)*

Bleeding from oesophageal varices, oesophageal ulcers or oesophagitis is occasionally massive and difficult to control. Octreotide, a synthetic analogue of somatostin lowers portal pressure and collateral blood flow including that through varices, increases lower oesophageal sphincter pressure, and inhibits the gastric secretion of acid as well as pepsin. Our current experience suggests it is effective in controlling acute variceal haemorrhage. Therefore we have examined the efficacy of octreotide in the control of post-sclerotherapy bleeding from oesophageal varices, oesophageal ulcers and oesophagitis. During the study period 77 patients experienced a significant gastrointestinal bleed (blood pressure < 100 mm Hg, pulse > 100 beats per min or the need to transfuse 2 or more units of blood to restore the haemoglobin level) following injection sclerotherapy of oesophageal varices. The source of bleeding was varices in 42 patients, oesophageal ulcers in 31 and oesophagitis in 4. All patients received a continuous intravenous infusion of octreotide (50 µg/h) for between 40–140h. If bleeding was not controlled in the first 12h after commencing octreotide hourly bolus doses (50 µg) for 24 h were superimposed on the continuous infusion. Haemorrhage was successfully controlled by an infusion of octreotide in 38 of the 42 patients with bleeding from varices, in 30 of 31 patients with oesophageal ulceration, and all patients with oesophagitis. In the 1 patient with persistent bleeding from oesophageal ulceration and in 2 of the 4 with continued haemorrhage from varices, haemostasis was achieved by hourly boluses of 50 µg octreotide for 24h in addition to the continuous infusion. No major complications were associated with octreotide administration. The results of this study clearly indicate that octreotide is a safe and effective treatment for the control of severe haemorrhage after technically successful injection sclerotherapy.

KEY WORDS: Octreotide haemorrhage oesophageal varices oesophageal ulcers oesophagitis injection sclerotherapy

### INTRODUCTION

Injection sclerotherapy is an effective treatment for the control of acute variceal bleeding and for subsequent obliteration of the varices. Localised rebleeding following injection sclerotherapy of oesophageal varices can occur from 3 sources, the varices themselves, oesophageal ulcers and oesophagitis. Persistent or early recurrent bleeding from the varices following injection sclerotherapy is often substantial, and is independently associated with a poorer prognosis[1]. In contrast, bleeding from oesophageal ulcers or oesophagitis after injection sclerotherapy is usually minor, but can occasionally be massive and difficult to control. We have recently demonstrated that somatostatin is a very effective and safe treatment for the control of recurrent bleeding following injection sclerotherapy from the varices[2], oesophageal ulcers and oesophagitis[3]. Octreotide, a synthetic analogue of somatostatin, has similar effects as the native hormone on portal pressure[4], azygos blood flow[5,6] intravariceal pressure[7], the gastric secretion of pepsin and acid[8] and on lower

*Correspondence to*: Dr. S.A. Jenkins, Academic Department of Surgery, Postgraduate Medical School, Morriston Hospital Swansea SA6 6NL.

oesophageal sphincter pressure[9]. These properties of somatostatin are thought to be the main mechanisms of action whereby the hormone is effective in controlling recurrent bleeding from varices, oesophageal ulcers or oesophagitis after sclerotherapy. We have therefore evaluated the efficacy of octreotide in the management of post-sclerotherapy bleeding.

### PATIENTS AND METHODS

Between January 1989 and December 1992, 225 patients presenting with acute variceal haemorrhage, were treated as an emergency, and 127 patients treated electively on 519 occasions by injection sclerotherapy. Injection sclerotherapy was carried out with a flexible endoscope using an intravariceal technique of 2–3 ml aliquots up to a maximum of 20 ml at each session. On some occasions however, the sclerosant was accidentally injected paravariceally. If oozing persisted after two 10 min periods of tamponade with the endoscope or overtube, a Sengstaken tube was inserted for 12 h. In patients with acute variceal haemorrhage, injection sclerotherapy was repeated 5–7 days later provided that there was no recurrent bleeding during the intervening period. Elective sclerotherapy was then carried out at 3–4 week intervals until the varices were obliterated.

During the study period 42 patients experienced significant recurrent bleeding from their varices, (within 24 h following sclerotherapy), 31 from oesophageal ulcers and 4 from oesophagitis. A significant haemorrhage was defined as either haematemesis and or melaena accompanied by either a systemic disturbance (systolic blood pressure less than 100 mm Hg and a heart rate greater than 100 beats/min) requiring blood tranfusion to maintain the vital signs, or the necessity to transfuse 2 or more units of blood to restore the haemoglobin levels. To the prehaemorrage levels. In all patients the site of haemorrhage was confirmed endoscopically, apart from those who bled in the first 24 h after sclerotherapy. In these patients the site of haemorrhage was assumed to be from the varices since oesophageal ulceration and oesophagitis after sclerotherapy take longer to develop. The absence of oesophagitis, oesophageal ulceration and other sources of recurrent bleeding determined by repeat endoscopy, carried out after cessation of octreotide therapy, confirmed the diagnosis of recurrent variceal haemorrhage. In no patient bleeding from oesophageal ulcers or oesophagitis could a variceal component to the haemorrhage be detected, but in deep lesions a venous component could not be excluded. The demographic data, grading of the severity according to the criteria of Childs'[10], aetiology, blood transfusion requirements, the duration of octreotide therapy and the time interval between the last sclerotherapy and onset of bleeding are shown in Table 1.

### *Octreotide treatment*

Initially, all patients with recurrent bleeding from varices, oesophageal ulcers or oesophagitis were treated with a continuous infusion of 50 µg/h octreotide. If bleeding was not controlled in the first 12 h of treatment the patients were given hourly bolus injections of octreotide (50 µg) for 24 h in addition to the continuous infusion.

### *Success of treatment*

Successful control of haemorrhage was defined as the cessation of bleeding, as confirmed by the absence of overt signs and the stabilisation of the haemoglobin levels, without the need for blood transfusion.

### RESULTS

### *Recurrent variceal bleeding*

During the study period a total of 744 courses of sclerotherapy were carried out, 225 to control an acute variceal bleed and 519 electively. Continued or early recurrent bleeding from varices occurred in 42 patients following sclerotherapy, an incidence of 5.6%. The incidence of recurrent variceal bleeding was much higher after emergency sclerotherapy (40/225; 18%) than following an elective session (2/519; 0.4%).

In the 42 patients who rebled following sclerotherapy the sclerosant had been administered intravariceally in 36 and paravariceally in 6 at the session preceding their recurrent haemorrhage. The volume of ethanolamine injected in patients who subsequently rebled (median 10, range 3–18 ml) was not significantly different from that administered to patients who did not rebleed (median 8.5, range 2–20 ml).

A continuous infusion of octreotide controlled persistent or recurrent variceal bleeding in 38/42 (90.5%) patients (Table 2). In four patients with severe liver disease (Child's C) bleeding was not controlled during the first 12 h of octreotide infusion as indicated by continued haematemesis and/or melaena accompanied by a systemic disturbance, and the need to

**Table 1** Demographic data, aetiology and severity of liver disease, duration of octreotide therapy and time interval between the last sclerotherapy in patients bleeding from varices, oesophageal ulcers or oesophagitis following sclerotherapy

| *Source of bleeding* | *Varices* | *Oesophageal Ulcers* | *Oesophagitis* |
|---|---|---|---|
| Age range (median) years | 21–73; 54.5 | 30–76; 59 | 63–79; 71 |
| Male/Female | 28:14 | 20:1 | 4:0 |
| Aetiology of liver disease | | | |
| Alcohol | 34 | 26 | 2 |
| Alcohol plus hepatocellular carcinoma | 1 | 0 | 0 |
| Cryptogenic | 3 | 2 | 2 |
| Chronic active hepatitis | 1 | 1 | 0 |
| Primary biliary cirrhosis | 2 | 1 | 0 |
| Idiopathic | 1 | 0 | 0 |
| Haemochromatosis | 0 | 1 | 0 |
| Child's grade | | | |
| A | 1 | 3 | 2 |
| B | 9 | 3 | 2 |
| C | 32 | 25 | 0 |
| Time to onset of bleeding after sclerotherapy (range;median) days | 0–72; 14.5 | 2–29; 9 | 10–171; 75 |
| Details of sclerotherapy prior to bleeding | | | |
| Volume of sclerosant (range:median) | 3–18; 10 | 4–18; 9 | 2–9; 8 |
| Intravariceal injections | 36 | 11 | 0 |
| Paravariceal injections | 0 | 1 | 2 |
| Mixed | 6 | 19 | 3 |
| Blood transfusion requirements (units) | | | |
| Before octreotide treatment (range:median) | 3–13; 3.5 | 3–9;5 | 4–8; 5.5 |
| During octreotide treatment (range:median) | 1–7; 2.5 | 1–4; 2 | 1–4; 2 |
| Duration of octreotide therapy h (range:median) | 40–140; 80 | 40–120; 80 | 80–140; 120 |

**Table 2** Efficacy of octreotide in controlling post-sclerotherapy bleeding from oesophageal varices, oesophageal ulcers and oesophagitis

| *Source of bleeding* | *Control of bleeding* | | *Hospital Mortality* |
|---|---|---|---|
| | *Octreotide infusion (50 μg/h)* | *Octreotide infusion plus hourly 50 μg bolus doses for 24h* | |
| Oesophageal varices | 38/42 (90.5%) | 2/4 | 8/42 (19%) |
| Oesophageal ulcers | 30/31 (97%) | 1/1 | 3/31 (9.7%) |
| Oesophagitis | 4/4 (100%) | | 0/4 |

transfuse blood to maintain the haemoglobin level. In two of these patients bleeding was controlled by giving hourly bolus doses of 50 μg octreotide in addition to a continuous infusion. Thus, overall control of bleeding was achieved in 40/42 (95%) patients treated with octreotide. The two patients who failed octreotide therapy continued to bleed despite further sclerotherapy and balloon tamponade, and haemorrhage was eventually controlled by emergency oesophageal transection. However, both patients developed hepatorenal syndrome following operation and died.

Recurrent bleeding did not occur in any of the 40 patients after cessation of octreotide therapy and 33 were discharged from hospital. Six patients, all with severe liver disease on admission and all presenting with an acute variceal bleed, died (liver failure n = 3, hepatorenal syndrome N = 3) in hospital. Thus, the overall hospital mortality was 8/42 (19%).

*Oesophageal ulceration*

Severe bleeding from oesophageal ulceration occurred in 31 patients, an overall incidence of 4.1% The incidence of severe bleeding from oesophageal ulceration was lower after a single course of sclerotherapy to control a variceal bleed (1/225; 0.4%) than after elective sclerotherapy (30/519; 5.8%). In all patients apart from one who bled 29 days after the previous session of sclerotherapy, the varices were patent at the diagnostic endoscopy, but in no case was a variceal component to the bleeding observed. However, in all these patients the possibility that there were communicating vessels between the ulcers and either the varices or the complex arrangement of blood vessels in or around the oesophagus cannot be discounted.

In the 31 patients who bled from oesophageal ulcers, the sclerosant was administered intravariceally in 11, paravariceally in 1 and both intravariceally and paravariceally in 19 at the sclerotherapy session preceeding their haemorrhage. The volume of sclerosant injected in the patients who subsequently bled from their oesophageal ulcers (median 9, range 2–20 ml) was not significantly different from that injected in patients who did bleed from oesophageal ulcers (median 8, range 2–19 ml).

A continuous infusion of octreotide (50 μg/h) controlled appreciable bleeding from oesophageal ulcers in 30/31 (97%) patients. In the one patient in whom bleeding persisted, control of haemorrhage was achieved by hourly bolus doses of 50 μg octreotide superimposed on their continuous infusion (Table 2). Bleeding did not recur in any of the patients following cessation of octreotide treatment, and 28 were discharged from hospital. However 3 patients, all with severe liver dysfunction (Child's C) and alcoholic liver disease died, one from liver failure and two from the hepatorenal syndrome.

### *Oesophagitis*

Appreciable bleeding from oesophagitis occurred only in 4 patients and in each case followed elective injection sclerotherapy. Thus the incidence of appreciable bleeding from oesophagitis after sclerotherapy was 0.54%.

In 3 patients, bleeding from oesophagitis after sclerotherapy occurred much later than that from the varices or oesophageal ulcers (Table 1). At endoscopy, 3 of the patients had obliterated varices, and one had a single small patent varix. All patients had undergone numerous courses of injection sclerotherapy (median 7, range 5–14). At the endoscopy prior to presenting with appreciable bleeding from circumferential oesophagitis affecting the distal 5cm of the oesophagus, the sclerosant was injected paravariceally in 1 and both intravariceally and paravariceally in 3. The amount of ethanolamine injected in the patients who bled from oesophagitis (median 8, range 2–9) was less than in those who did not bleed from oesophagitis (median 10, range 2–20). Early rebleeding after sclerotherapy (10 days) occurred in one patient who had received only paravariceal administration of sclerosant at the endoscopy prior to the development of haemorrhagic oesophagitis.

Octreotide infusion successfully controlled the bleeding in all 4 patients bleeding from oesophagitis (Table 2). No further bleeding occurred after cessation of octreotide treatment and all 4 patients made an uneventful recovery and were discharged home on ranitidine and gaviscon.

### DISCUSSION

The incidence of recurrent variceal bleeding following injection sclerotherapy observed in this study is similar to that of previously reported series[11]. Balloon tamponade is widely used to control recurrent variceal bleeding following sclerotherapy. However, balloon tamponade is associated with serious side effects, including oesophageal ulceration and perforation in 10–25% of patients when used as a stop-gap therapy to control bleeding before definitive therapy[12]. Following sclerotherapy, the risk of balloon tamponade producing perforation or ulceration of the oesophagus may be increased, because of the damaging effects of the sclerosant on the oesophageal mucosa[13]. Consequently, it has been suggested that the period of balloon tamponade should be limited to a period of 12 h after sclerotherapy to minimise the risk of local complications[12]. If bleeding persists following a 12 h period of balloon tamponade treatment can be problematical. Further periods of tamponade may control bleeding but the complication rate can become unacceptably high. Similarly, a second course of sclerotherapy may control bleeding, but in those patients in which it is not successful, the mortality approaches 100%[14]. Furthermore emergency oesophageal transection is often unrewarding because of difficulty in mobilising the oesophagus after injection sclerotherapy[14] and the high mortality associated with surgical intervention in patients with poor liver function[15]. We have recently demonstrated that somatostatin is a very effective and safe treatment for the control of recurrent variceal bleeding following sclerotherapy[2]. The results of the present study in a much larger number of patients suggest that octreotide is equally as effective as somatostatin in controlling post-sclerotherapy variceal bleeding. Furthermore, no complications were associated with octreotide therapy. In 2 of the 4 patients in whom octreotide failed to control haemorrhage, haemostasis was achieved by administering hourly bolus doses of octreotide for 24 h, in addition to the continuous infusion. The rationale for this treatment was based on observations in the portal hypertensive cirrhotic rat which suggested that bolus administrations of somatostatin reduced portal pressure and collateral blood flow to a greater extent than a continuous infusion[17,18], observations recently confirmed in man[19]. Therefore, bolus injections of octreotide together with a continuous infusion of octreotide, could possibly reduce collateral blood flow to a greater extent than an infusion alone and thus facilitate control of bleeding. This suggestion is supported to some extent in this study, since 2 of the 4 patients stopped bleeding during their treatment with hourly bolus doses of octreotide superimposed on a continuous infusion.

Severe bleeding from oesophageal ulcers or oesophagitis following injection sclerotherapy is perhaps more problematical than that of recurrent variceal haemorrhage, since there are fewer treatment options available. Balloon tamponade is contraindicated in these circumstances since pressure necrosis may exacerbate the ulceration or oesophagitis, whilst oesophageal transection is difficult and unrewarding in patients with poor liver function[15,16]. Consequently, effective drug therapy is required to control bleeding from these sources. We have demonstrated that somatostatin is a very effective and safe treatment for the control of severe bleeding from post-sclerotherapy oesophageal ulceration and oesophagitis[3]. The results of this study indicate that an infusion of 50 μg/h octreotide is equally as effective as somatostatin in these indications, controlling bleeding in all 4 patients with oesophagitis and 30/31 patients with oesophageal ulceration. Furthermore, in the patient with oesophageal ulcers who continued to bleed during octreotide infusion, addition of hourly boluses of the somatostatin analogue for 24 controlled haemorrhage. This observation further suppors the suggestion that bolus doses of octreotide superimposed on a continuous infusion may be useful in controlling persistent bleeding, possibly by reducing collateral blood flow to a greater extent than an infusion alone. No major side-effects were associated with octreotide administration, with the exception of one patient who experienced a marked increase in the plasma levels of bilirubin and liver enzymes. Following oessation of octreotide administration in this patient the plasma levels of bilirubin and liver enzyme gradually returned to pre-treatment levels.

Although reports on the effects of octreotide on portal pressure have provided conflicting results, the analogue has been consistently demonstrated to lower azygos blood flow[5,6]. Furthermore, octreotide reduces azygos blood flow to a greater extent than it reduces portal pressure[5,18]. Consequently, the reduction in azygos blood flow, indicative of a reduction in collateral blood flow including that through the varices, may be the most important determinant in controlling variceal bleeding. A reduction in collateral blood flow, including that through the complex venous network in the lamina propria of the oesophagus, may also be a major factor in controlling bleeding from post-sclerotherapy ulcers and oesophagitis. In portal hypertension, the 4 layers of veins of the lower oesophagus are grossly dilated, and the large intrinsic vessels which form the varices communicate directly or indirectly with all the remaining venous channels[20]. It is therefore impossible to exclude a venous component to the haemorrhage from oesophagitis or oesophageal ulcers after sclerotherapy, since it seems likely that there are communications between these lesions, and other venous components of the oesophagus or peri-oesophageal veins. Thus although bleeding could be observed from oesophageal ulcers or from the friable mucosa in oesophagitis and not from the varices, it is not possible to exclude a venous component to the haemorrhage, and a reduction in blood flow through these vessels elicited by octreotide may be an important factor in controlling haemorrhage from these lesions. The precise mechanism whereby octreotide decreases collateral blood flow is unknown, but may be mediated directly by eliciting vasoconstriction through specific receptors in these vessels[21]. Alternatively, octreotide may reduce collateral blood flow via inhibition of the release of vasoactive substances responsible for the hyperdynamic circulation in portal hypertensive patients[21]. Finally, by increasing lower oesophageal pressure[9] octreotide may decrease blood flow through complex venous network of the lower oesophagus.

Following initial control of haemorrhage, octreotide has other effects which may be beneficial in preventing recurrent bleeding. Although erosion of the varices by reflux of gastric contents is not thought to contribute to variceal bleeding *per se*, it may, by dissolution of the fibrin plug or clot at the site of haemostasis, precipitate further bleeding. Octreotide by inhibiting the secretion of gastric acid and pepsin[8] and by increasing lower oesophageal sphincter pressure, may reduce the volume and digestive capacity of any refluxed material, and thereby minimise risk of clot or fibrin plug dissolution and hence rebleeding.

In summary, the results of this study that octreotide is an effective treatment for post-injection sclerotherapy bleeding from oesophageal varices or ulcers and oesophagitis. These encouraging observations require confirmation in randomised controlled trials.

*Acknowledgements*

This study was supported by a grant from the Mersey Regional Health Authority and Sandoz Pharmaceuticals U.K.

The results of this study have been presented to the Surgical Research Society and the British Society of Gastroenterology and published in abstract form in the British Journal of Surgery and Gut.

### REFERENCES

1. Burroughs A.K, D'Heygere F, Phillips A, McIntyre N. (1986) Predictive model for early failure to control variceal bleeding. *Hepatology*, **6**: 115 Abs.
2. Jenkins S.A, Shields R, Jaser N, Ellenbogen S, Naylor E. & Baxter JN. (1992) The management of persistent or recurrent variceal bleeding after injection sclerotherapy by somatostatin. *HPB Surg*, **5**: 221-227.
3. Jenkins S.A, Shields R, Jaser N, Ellenbogen S, Makin C, Naylor E, *et al.* (1991) The management of gastrointestinal haemorrhage by somatostatin after apparently successful endoscopic injection sclerotherapy for bleeding oesophageal varices. *J Hepatology*, **12**: 296-301.
4. Hamisch E, Doertenbach J, Usadel K.H. (1992) Somatostatin in acute bleeding oesophageal varices. *Drug*, **44** (Suppl 2): 24–35.
5. Navasa M, Bosch J, Chesta J, Bru C, Piccueta P, Garcia-Pagan J.C *et al.* (1988) Haemodynamic effects of subcutaneous administration of SMS 201-995, a long acting analogue of somatostatin in patients with cirrhosis and portal hypertension. *J Hepatol*, **7** (Suppl): S64 Abst.
6. McCormick P.A, Dick R, Siringo S, Wagstaff D, Chesta J, McIntyre N, Burroughs A.K. (1990) Octreotide reduces azygos blood flow in cirrhotic patients with portal hypertension. *Eur J Gastroenterol Hepatol*, **2**: 489–498.
7. Jenkins S.A, Baxter J.N, Corbett W.A & Shields R. (1985) Effects of a somatostatin analogue SMS 201- 995 on hepatic haemodynamics in the pig and on intravariceal pressure in man. *Br J Surg*, **72**L 1009–1012.
8. Whitehouse I, Bollinger C, Freud M, Gyr K. (1984) The effect of an octopeptide somatostatin analogue (SMA 201- 995) and somatostatin (SST-14) on pentagastrin stimulated gastric acid secretion. A comparative study in man. *Hepatogastroenterology*, **31**: 227–229.
9. Gunshefski L.A, Ripley W.J, Glattery D.W, Scwiffini J, Harsuck M & Little A.G. (1992) Somatostatin stimulation of the normal esophagus. *Am J Surg*, **143**: 59–62.
10. Child CG, Turcotte J.G. Surgery and portal hypertension. In: Child G, Dunphy J.E (Eds). (1964) Major problems in clinical surgery. The liver and portal hypertension. Vol 1. Philadelphia, *WB Saunders*, 1–85.
11. Graham D.Y, Smith J.L. (1981) The course of patients after variceal haemorrhage. *Gastroenterology*, **80**: 800–809.
12. Blavianos P, Gimson A.E.S, Westaby D, Williams R. (1989) Balloon tamponade in variceal bleeding: Use and misuse. *Br Med J*, **298**: 1158.
13. Evans D.M.D, Jones D.B, Clearly B.K, Smith P.M. (1982) Esophageal varices treated with sclerotherapy: a histopathologic study. *Gut*, **23**: 615–620.
14. Terblanche J, Burroughs A.K & Hobbs K.F. (1989) Controversies in the management of bleeding oesophageal varices. *New Engl J Med*, **320**: 1393–1398.
15. Johnson A.G. (1982) Oesophageal transection and devascularisation procedures. In Westaby D, MacDougall B.R.D & Williams R.D. Eds. Variceal Haemorrhage, London, Pitman : 97–103.
16. Jenkins S.A, Shields R. (1988) Variceal haemorrhage after failed injection sclerotherapy: The risk of emergency oesophageal transection. *br J Surg*, **76**: 49–51.
17. Jenkins S.A, Devitt P, Day D.W, Baxter J.N & Shields R. (1986) The effects of somatostatin on hepatic haemodynamics in the cirrhotic rat. *Digestion*, **33**: 161–168.
18. Jenkins S.A, Yates J, Nott D.M, Ellenbogen S, Cooke T.G & Shields R. (1989) Effects of vasoactive drugs on the collateral circulation of rats with portal hypertension. *J Roy Coll Surg Edin*, **24**: 273–274.
19. Neuens F, Sprengeres D, Feberty J. (1994) The effects of different doses of a bolus injection of somatostatin combined with a slow infusion of transmural oesophageal variceal pressure in patients with cirrhosis. *J Hepatol*, **20**: 27–31.
20. Kitano S, Terblanche J, Kahn D, Bornman PC. (1986) Venous anatomy of the lower oesophagus in portal hypertension. practical Implications. *Br J Surg*, **83**: 525–531.
21. Jenkins S.A. (1992) Somatostatin in acute bleeding oesophageal varices. Clinical evidence. *Drugs*, **44** (Suppl 2): 36–55.

